# The complete chloroplast genome of *Cirsium setosum* and its phylogenetic analysis

**DOI:** 10.1080/23802359.2019.1681921

**Published:** 2019-10-24

**Authors:** Binxin Xie, Chunyan Jiang, Ziyan Zhu, Mingzhi Li, Yucheng Zhao

**Affiliations:** a Department of Resources Science of Traditional Chinese Medicines and State Key Laboratory of Natural Medicines, School of Traditional Chinese Pharmacy, China Pharmaceutical University, Nanjing, Jiangsu, P. R. China;; b Jinling Hospital, Nanjing, Jiangsu, P. R. China;; c Biodata Biotechnology Co. Ltd, Heifei, Anhui, P. R. China

**Keywords:** *Cirsium setosum*, complete chloroplast genome, phylogenetic analysis

## Abstract

*Cirsium setosum* is a widely distributed species of edible medicinal plant around the world. Triterpenes, flavonoids, sterols, polyphenols, and glycosides are its main medicinal ingredients. In this study, the complete chloroplast genome sequence of *C. setosum* was assembled and characterized from high-throughput sequencing data. The chloroplast genome was 152,405 bp in length, consisting of large single-copy (LSC) and small single-copy (SSC) regions of 83,385 bp and 18,632 bp, respectively, which were separated by a pair of 25,193 bp inverted repeat (IR) regions. The genome is predicted to contain 133 genes, including 88 protein-coding genes, 37 tRNA genes, and 8 rRNA genes. The overall GC content of the genome is 37.7%. A phylogenetic tree reconstructed by 12 chloroplast genomes reveals that *C. setosum* is mostly related to *Cirsium arvense,* which is also a Cephalanoplos plant widely distributed all over the world. The work reported the firstly complete chloroplast genome of *C. setosum* which may provide useful information to the evolution of Cephalanoplos.

*Cirsium setosum* is an edible medicinal plant that is widely distributed around the world. It is an important medicinal component in traditional Chinese medicine for many years, which was supported by considerable clinical evidence (Ma et al. 2016a). For instance, *C. setosum* is widely used to treat many types of diseases, including incomplete uterine contraction and bleeding, infectious hepatitis, digestive tract bleeding, cardiovascular disease, and promote aggregation and adhesion of platelets (Lu et al. 2015; Ma et al. 2016a). Modern phytochemical studies revealed that triterpenes, flavonoids, sterols, polyphenols, and glycosides are its main medicinal ingredients (Luan et al. 2016; Ma et al. 2016b; Ma, Wen, et al. 2016). However, little is known in its biosynthesis mechanism. In addition, the phylogenetic position of *C. setosum* and the genus Cephalanoplos is still unresolved. In this study, we first reported the complete chloroplast genome of *C. setosum*, its phylogenetic analysis is also investigated which provide informatics data for the phylogeny of genus Cephalanoplos.

The fresh leaves of *C. setosum* from Nanjing, Jiangsu, China (31°54′N, 118°54′E) were used for genomic DNA extraction. Specimens were stored in Department of Resources Science of Traditional Chinese Medicines of China pharmaceutical University with the accession number of XJ20190615XBX-2. Total genomic DNA was extracted with a FastPure Plant DNA Isolation Mini Kit (Vazyme, Nanjing, China). The whole genome sequencing was conducted by Hefei Biodata Biotechnologies Inc. (Hefei, China) on the Illumina Hiseq platform. The filtered sequences were assembled using the programme SPAdes assembler 3.10.0 (Anton et al. 2012). Annotation was performed using the DOGMA and BLAST searches (Wyman et al. 2004). The cp genome of *C. setosum* was determined to comprise a 152,403 bp double-stranded, circular DNA (GenBank accession no. MN432154), containing two inverted repeat (IR) regions of 25,193 bp, separated by large single-copy (LSC) and small single-copy (SSC) regions of 83,385 bp and 18,632 bp, respectively. The overall GC content of *C. setosum* cp genome is 37.7% and the corresponding values in LSC, SSC, and IR regions are 35.9%, 31.4%, and 43.1%, respectively. The cp genome was predicted to contain 133 genes, including 88 protein-coding genes, 37 tRNA genes, and 8 rRNA genes. Six protein-coding genes, six tRNA genes, and four rRNA genes were duplicated in IR regions. Nineteen genes contained two exons and four genes (clpP, ycf3 and two rps12) contained three exons.

To investigate its taxonomic status, alignment was performed on the 12 chloroplast genome sequences using MAFFT v7.307, and a maximum likelihood (ML) tree was constructed by FastTree version 2.1.10 (Price et al. 2010; Kazutaka and Standley 2013). As expected, *C. arvense* is the mostly related species to *C. setosum*, with bootstrap support values of 100% (Figure 1). The complete cp genome sequence of *C. setosum* will provide a useful resource for the conservation genetics of this species as well as for the phylogenetic studies of Cephalanoplos.

**Figure 1.**
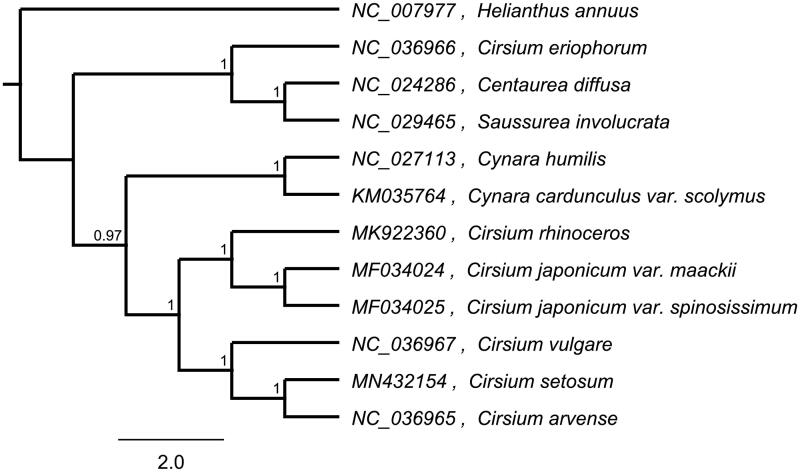
Phylogenetic tree inferred by maximum likelihood (ML) method based on 12 representative species. Helianthus annuus was used as outgroup. A total of 1000 bootstrap replicates were computed and the bootstrap support values are shown at the branches. GenBank accession numbers are shown in the figure.
